# Clinical advances in oncolytic virus therapy for malignant glioma: a systematic review

**DOI:** 10.1007/s12672-023-00769-1

**Published:** 2023-10-16

**Authors:** Shan Jiang, Huihui Chai, Qisheng Tang, Zhifeng Shi, Liangfu Zhou

**Affiliations:** 1 National Center for Neurological Disorders, Shanghai, China; 2 Department of Neurosurgery, Huashan Hospital, Fudan University, Shanghai, China; 3 Institute of Neurosurgery, Fudan University, Shanghai, China; 4 Shanghai Clinical Medical Center of Neurosurgery, Shanghai, China

**Keywords:** Oncolytic virus, Malignant glioma, Clinical trials, Systematic review

## Abstract

**Purpose:**

In the past decade, there has been little progress in the treatment of malignant glioma. Recently, oncolytic virus has made great progress in glioma treatment, and a number of clinical trials have shown their potential of prolonging the survival time of glioma patients. Our objective is to evaluate effectiveness and safety of oncolytic virus (OV) in malignant glioma treatment.

**Methodology:**

Based upon PRISMA, we collected relevant published clinical trials by searching medical databases up to January 16, 2023, applying the language restrictions in English and Chinese. We cross-searched the terms: ‘glioma’, ‘glioblastoma’, ‘oncolytic viruses’, ‘oncolytic virotherapy’ with filter ‘clinical trial’. Two researchers independently extracted the data regarding case definitions, published years, trial phase, characteristics of patients, administration of drug, overall survival (OS), and adverse events.

**Results:**

19 published clinical trials in OV treatment of malignant glioma were included in the further systematic review analysis. None of them induced irresistible adverse effects attributing to OV treatment, median overall survival varied from 3.25 to 20.2 months after treatments. According to trials providing patient’s detailed molecular diagnosis, we find that the effectiveness of OV treatment has no significant difference in patients with different IDH or MGMT status.

**Conclusions:**

Current clinical trials have initially shown the potential of oncolytic virotherapy as a new treatment for malignant glioma. Besides development of virus types, the strategy of OV use is an urgent problem to be solved in future clinical application, such as repeated administrations, innovative drug delivery systems, and biomarkers.

## Background

Malignant glioma accounts for nearly 80% of all malignant primary brain tumors [1]. The annual incidence of glioma is 3–6.4/100,000, The median survival time of glioblastoma is less than 20 months, and the 5-year survival rate is only about 5.8% after standard combined treatment. At present, the proven treatment for malignant glioma is surgical resection, chemotherapy, radiotherapy, and electric field therapy, while there are no effective therapies for recurrent glioblastoma. The past decades have witnessed the developments of CAR-T therapy, tumor vaccine therapy, and oncolytic virus therapy, making immunotherapies the most potential ones for treatment of recurrent malignant glioma.

Among these immunotherapies, oncolytic virus showed great potential for clinical application. Currently, several virus species were performed in the trials of malignant glioma treatment, such as Herpes Simplex Virus‑1, Adenovirus, Reovirus, Parvovirus, Polio/Rhinovirus Recombinant, Newcastle Disease Virus, and so on. Oncolytic viruses can be natural or genetically modified viruses which has the capability to replicate in the tumor, and their antitumor activity involves a variety of mechanisms [2]. Firstly, the oncolytic viruses could specifically infect into tumor cells and elicit tumor lysis by viral replication and antiviral innate immunity. The specific infection is due to its biological habits or artificially added selective promotor [3, 4]. Secondly, the tumor lysis therefore induces the release of tumor associated antigens (TAAs), cell-derived damage-associated molecular patterns (DAMPs) and viral pathogen-associated molecular patterns (PAMPs). The innate immune response including recruiting dendritic cells and lymphoid cells eliminates virus-infected tumor cells [5]. Thirdly, based on the above two processes, the spread and replication of oncolytic virus in tumor tissue change the tumor immune microenvironment from cold into hot, and promotes the body to produce anti-tumor immunity [6, 7]. Moreover, certain oncolytic adenoviruses inhibit brain cancer stem cells [8, 9]. The combined anti-tumor mechanisms of the oncolytic virus led to short-term and long-term anti-tumor efficacy, which is expected to improve the survival time of patients with malignant glioma. On June 11, 2021, oncolytic therapy Delytact (Teserpaturev/G47Δ) has been approved by the Ministry of Health, Labor and Welfare of Japan, making it the world’s first conditional and time-limited approved oncolytic viral therapy for brain tumours [10].

Considering the effectiveness of oncolytic virus therapy varies greatly, the purpose of this review is to systematically analyze the published clinical trials based on the evaluation of virus types, administration, efficacy and safety.

## Method

### Search strategy and selection criteria

This systematic review is conducted following the Preferred Reporting Items for Systematic Reviews and Meta-Analyses (PRISMA) 2020 Statement [11]. We collected relevant published clinical trials by searching PubMed, Embase, Web of Science, Cochrane Central Register of Controlled Trials (CENTRAL), ClinicalTrials.gov, China national knowledge infrastructure (CNKI), and Chinese Clinical Trial Registry (ChiCTR) up to July 20, 2023. Language restrictions in English and Chinese were applied. We cross-searched the terms: ‘glioma’, ‘glioblastoma’, ‘oncolytic viruses’, ‘oncolytic virotherapy’ with filter ‘clinical trial’.

We regarded studies as eligible for inclusion done in clinical trials studying oncolytic therapy in treatments of malignant glioma no matter in adults or children. Exclusive criteria were as follows: the study therapy that virus was only used as the vector to deliver therapeutic agent was excluded; the case reports, case series, and conference papers were excluded as well. Study titles and abstracts were reviewed by two independent investigators. Then the inclusion criteria were received after a full-text assessment. In case of disagreement, a third reviewer independently assesses the search methods, and any issue was resolved by consensus.

### Data collection and analysis

Under the guidance of the established research protocol, two researchers independently extracted the data regarding case definitions, published years, trial phase, characteristics of patients, administration of drug, overall survival (OS), and adverse events. Notably, we did not sum up the median progression-free survival because the main indicator of tumor progression was imaging evidence. While because of the intratumoral injection, the entire tumor enlargement might confuse the judgment of tumor progression. Moreover, Tomoki Todo et al. assumed that post-administration MRI features (injection site contrast-enhancement clearing and entire tumor enlargement) likely reflected tumor cell destruction via viral replication and lymphocyte infiltration towards tumor cells based on biopsies [12]. Finally, the types of viruses, drug administration methods, efficacy and side effects were analyzed and reviewed in this paper.

## Results

**Fig. 1:**
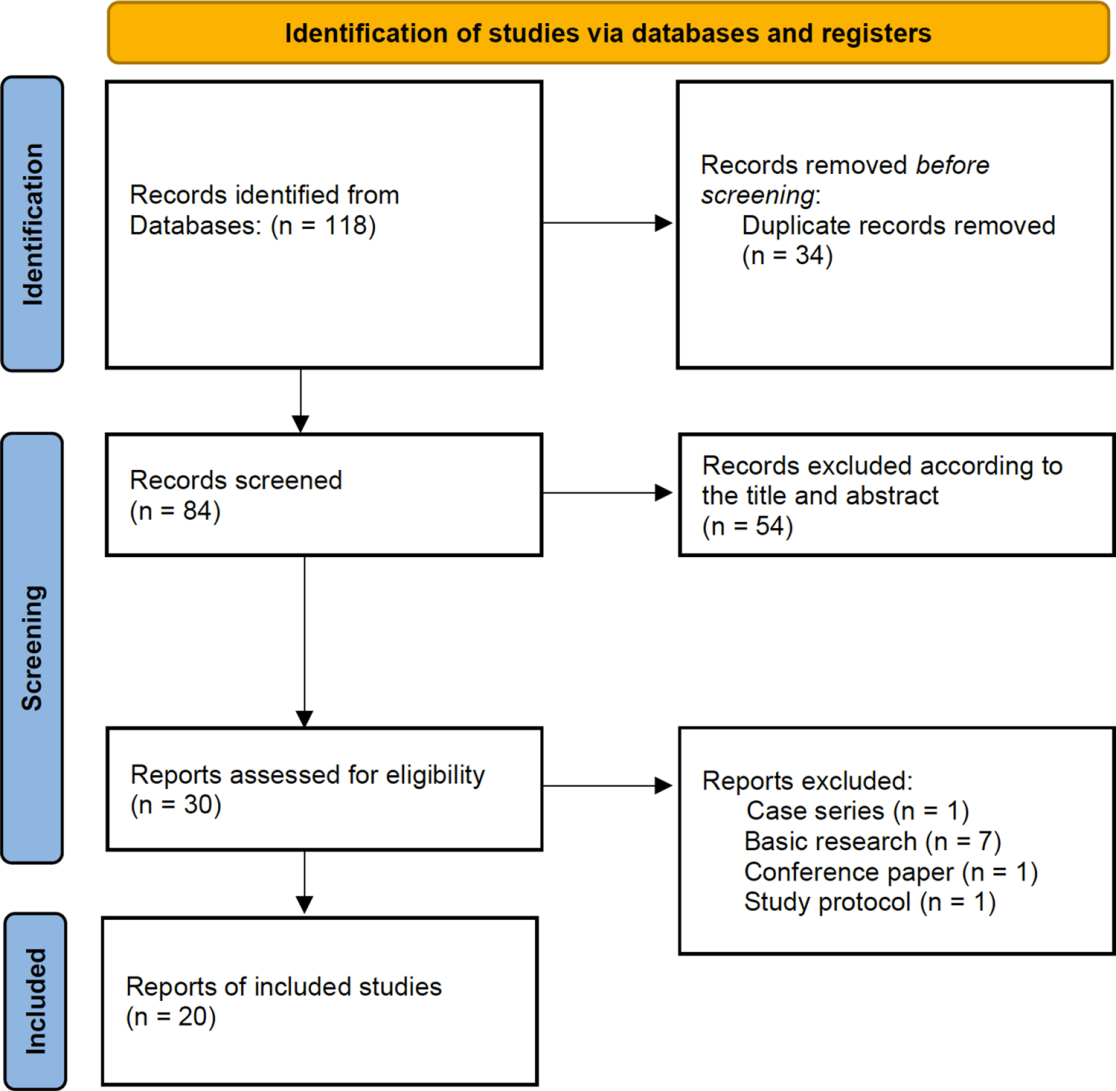
Flow diagram of database search and study identification

118 records were searched out from the medical databases mentioned above and 34 of them were excluded due to the duplication. After titles and abstracts screened, there were 30 published articles relevant and retrieving for full-text assessments: 10 of these studies were excluded according to the reasons listed in Fig. 1. Finally, 20 studies fully met the criteria and were included in the further systematic review analysis. The flow diagram of the research was summarized in Fig. 1. 15 of total 20 trials were in phase I, while rest 5 trials were in phase I/II or phase II. None of them staged in the phase III.

### Virus types

**Fig. 2:**
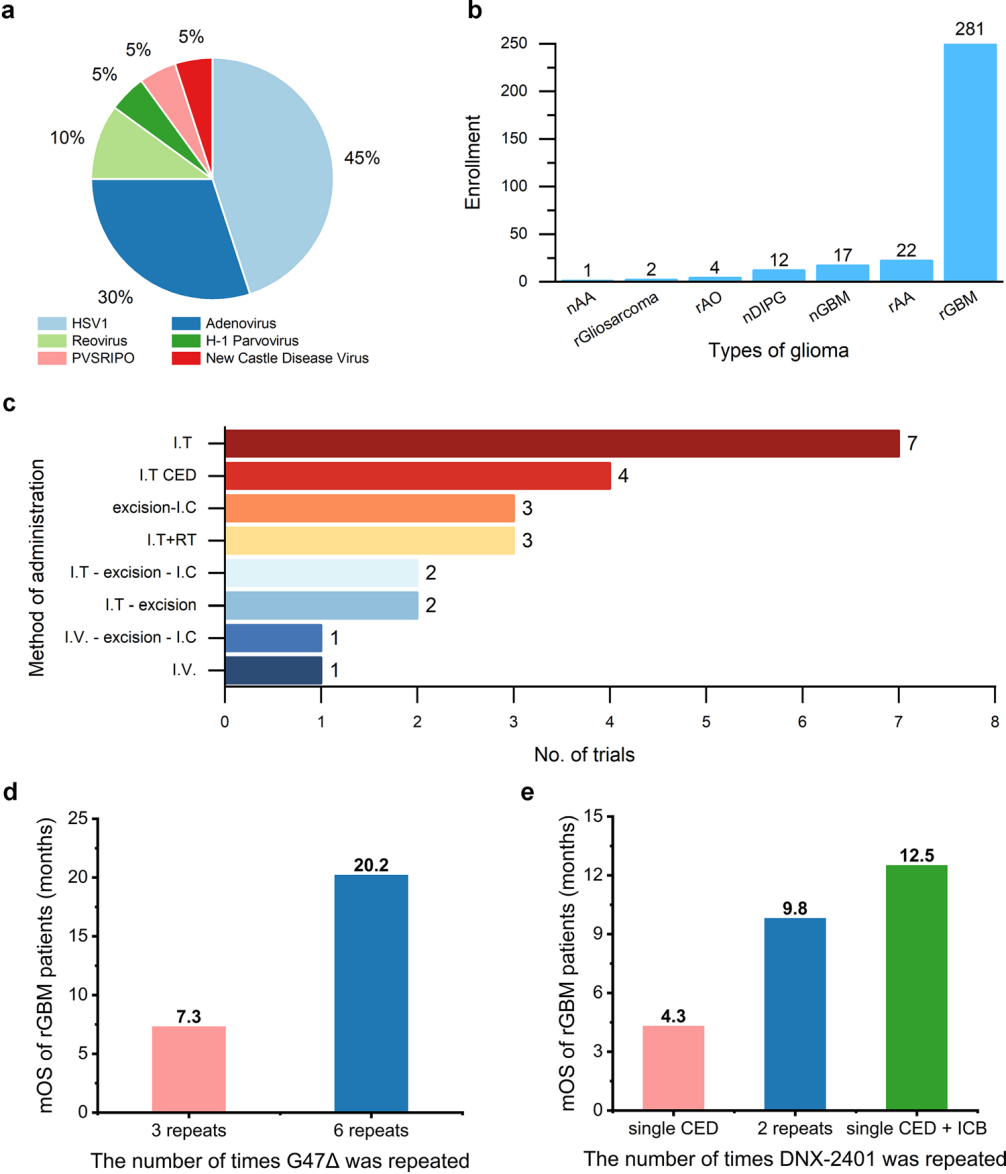
The distribution and characteristics of included clinical trials of oncolytic treatments for malignant gliomas. **a** The proportion of virus types used in the included oncolytic clinical trials. **b** The accumulative number of enrolled patients with different malignant glioma types in clinical trials. ‘r’ represents recurrent tumor and ‘n’ represents new diagnosis tumor. AA represents anaplastic astrocytoma, AO represents anaplastic oligodendroglioma, DIPG represents diffuse intrinsic pontine glioma, GBM represents glioblastoma multiforme; **c** the number of trials using different administrations to apply the virus. Certain trials set different subgroups with various administrations. Each of these subgroups was counted individually. **d** Comparison of median survival of rGBM patients with different dosing times in G47Δ clinical trials. **e** Comparison of median survival of rGBM patients with different administrations in DNX-2401 clinical trials

The majority of articles published to report the results of their clinical trials used HSV-1 (n = 9, 45%) and adenovirus (n = 6, 30%). There were 2 trials utilizing reovirus (10%), while Polio/Rhinovirus Recombinant virus (n = 1, 5%), H-1 Parvovirus (n = 1, 5%), and New Castle Disease virus (n = 1, 5%) has only one completed trial, respectively (Fig. 2a). The types of viruses used in the treatment reflected the multitudinous research and development route. Through virus transformation technology [13], the development of oncolytic virus products with different characteristics to achieve better glioma tropism and tumor-killing ability. We summarized and analyzed the use of different oncolytic viruses in published clinical studies, especially the maximum dose used and adverse events attributed to oncolytic therapy (Table 1).

**Table 1:** Published clinical trials of oncolytic therapy for malignant glioma

Year	Phase	Generic name	Glioma type	Administration	Max-dose	mOS	Adverse events (attributing to OV)	References
HSV-1								
2000	I	G207	rGBM:16 rAA:5	I.T	3 × 10^9^ PFU	6.2	There was no AE attributing to OV treatment	Markert et al. [14]
2000	I	HSV1716	rGBM:8 rAA:1	I.T	1 × 10^5^ PFU	9	There was no AE attributing to OV treatment	Rampling et al. [15]
2002	I	HSV1716	rGBM:11 rAA:1	I.T	1 × 10^5^ PFU	6.5	There was no AE attributing to OV treatment	Papanastassiou et al. [16]
2004	I	HSV1716	rGBM:4 rAA:1 rAO:1 nGBM:6	I.C	1 × 10^5^ PFU	11.25	There was no AE attributing to OV treatment	Harrow et al. [17]
2009	Ib	G207	rGBM:6	I.T *2	1.15 × 10^9^ PFU	6.6	II: fever III: seizure, decreased mental status, increased temperature (max 39.7 °C), hemiparesis, neglect, intermittent seizure activity, somnolence, motor neuropathy, sided weakness	Markert et al. [18]
2014	I	G207	rGBM:7 rAA:2	I.T+RT	1.0 × 10^9^ PFU	7.5	III: seizure, fever	Markert et al. [19]
2021	I	G207	rGBM:11 rAA:1	I.T+RT	1 × 10^8^ PFU+5 Gy	12.2	I: diarrhea, nausea, vomiting, chills, fatigue, fever, anorexia, dizziness, headache, seizure, hemorrhage	Friedman et al. [20]
2022	II	G47Δ	rGBM:19	I.T *6	1 × 10^9^ PFU	20.2	I: fever, vomiting, nausea, cerebral edema, neuropathy–sensory, headache, anemia, lymphocyte count decreased, white blood cell count decreased, platelet count decreased, bilirubin increased, γ-Glutamyl transpeptidase increased, PT-INR increased, hypoalbuminemia, hyponatremia II: fever, fatigue, vomiting, nausea, seizure, cerebral edema, lymphocyte count decreased, white blood cell count decreased, neutrophil count decreased III: fever, vomiting, headache, lymphocyte count decreased, white blood cell count decreased, neutrophil count decreased IV: lymphocyte count decreased	Todo et al. [10]
2022	I/II	G47Δ	rGBM:13	I.T *2	1 × 10^9^ PFU	7.3	I: fever, headache, nausea, vomiting, decreased hemoglobin, hemorrhage with surgery II: vomiting, seizure, fever III: decreased leukocytes	Todo et al. [12]
Adenovirus								
2004	I	ONYX- 015	rGBM:17 rAA 5 rAO 2	I.C	1 × 10^10^ PFU	6.2	There was no AE attributing to OV treatment	Chiocca et al. [21]
2018	I	DNX-2401	rGBM:33 rAA:2 rGliosarcoma:2	I.T/I.T-excision-I.C *2	3 × 10^10^ VP	13	I: headache, nausea, confusional state, vomiting, pyrexia	Lang et al. [22]
2021	I	NSC-CRAd-S-pk7	nGBM:11 nAA:1	I.C	1.875 × 10^11^VP/ 1.50 × 10^8^ NSC	18.4	II: subdural fluid collection III: meningitis	Fares et al. [23]
2022	I	DNX-2401	nDIPG:12	I.T+RT	5 × 10^10^ PFU	17.8	I: vomiting, fever, nausea, trigeminal nerve disorder, dizziness, facial nerve disorder II: fever, somnolence, headache, nystagmus III: neurological deterioration	Larraya et al. [24]
2022	I	DNX-2401	rGBM:19	I.T CED	1 × 10^11^ VP	4.3	Only reported the relationship of SAE with treatment: III: seizure, increased intracranial pressure, meningitis with hydrocephalus, increased intracranial pressure IV: confusion	Putten et al. [25]
2023	I/II	DNX-2401	rGBM:49	I.T+ pembrolizumab	5 × 10^10^ VP	12.5	I: brain edema, headache, fatigue, dysphasia, pyrexia, decreased appetite, myalgia, nausea II: brain edema, headache, fatigue, dysphasia, hemiparesis, decreased appetite, myalgia, nausea III: brain edema, headache, hemiparesis IV: brain edema	Nassiri et al. [26]
Reovirus								
2008	I	Reovirus	rGBM:9 rAA:2 rAO:1	I.T-excision	7 × 10^8^ PFU	5.25	III: GGT increasing	Forsyth et al. [27]
2014	I	Reovirus	rGBM:15	I.T CED	1 × 10^10^ PFU	4.6	There was no AE attributing to OV treatment	Kicielinski et al. [28]
H-1 parvovirus								
2017	I/IIa	ParvOryx	rGBM:18	I.T-excision-I.C/I.V-excision-I.C *2	5 × 10^9^ PFU	15.5	There was no AE attributing to OV treatment	Geletneky et al. [29]
Recombinant poliovirus								
2018	I	PVSRIPO	rGBM:61	I.T CED	7 × 10^9^ PFU	12.5	I: blurred vision, diplopia, focusing difficulty, visual field cut or hemianopia, nausea, vomiting, fatigue, gait disturbance, cognitive disturbance, dystonia, facial muscle weakness, headache, intracranial hemorrhage, paresthesia, pyramidal tract syndrome, seizure, confusion, hallucinations, urinary incontinence II: visual field cut or hemianopia, fatigue, cognitive disturbance, dysphasia, headache, pyramidal tract syndrome, seizure, confusion, vascular disorder, hypertension III: gait disturbance, dystonia, headache, pyramidal tract syndrome, seizure, confusion, delusions IV: cerebral edema V: seizure	Desjardins et al. [30]
Newcastle disease virus								
2006	I/II	NDV-HUJ	rGBM:11	I.V. *3	5.5 × 10^10^ EID50	8	I: fever II: fever	Freeman et al. [31]

### Administrations

All clinical trials were diagnosed due to WHO CNS4 classification because of the timing of trial initiation. Glioma subtypes enrolled in the trials were demonstrated in Fig. 2B. The most frequent administration of oncolytic viruses was direct intratumoral injection (I.T) (n = 12). Three of them were combined with radiotherapy [19, 20, 32]. Three trials excised the tumor body and then injected the virus into residual tumor cavities (I.C) [17, 21, 23]. The other four trials injected the virus into the tumor utilizing convection-enhanced delivery (I.T CED) [25, 26, 28, 30]. One of them began using pembrolizumab 7 days after intratumoral injection [26]. One trial performed the oncolytic therapy by venous transfusion using the New Castle Disease virus [31]. Several trials set different subgroup to compare different combinations among I.T, I.V, and I.C as we listed detailly in Table 1 [22, 33]. Meanwhile, the repeating times of virus administration in trials varied. 4 of them performed twice virus treatments [12, 18, 22, 29]. One trial was conducted with three doses [31]. The phase II trial of G47Δ carried out six-time virus injections [10]. It is worth mentioning that it is allowed to perform palliative resection, chemotherapy, radiotherapy, and any other necessary treatments for patients after receiving the oncolytic therapy. We only counted the designed standard administrations in the trials.

### Adverse events

The majority of adverse events that may be attributed to oncolytic virotherapy were not more than grade III. And most of symptoms are self-limited. The most common adverse events were fever and seizure. Only trial of PVSRIPO [34] reported a seizure of grade V. Total 4 trials reported grade IV adverse events such as cerebral edema [26, 34], confusion [25], and lymphocyte count decreased [10]. Specifically, because of the inadvertent injection of NSC-CRAd-Spk7 into the ventricle, oncolytic adenovirus CRAd-S-pk7 carried with neural stem cells caused a meningitis of grade III. After hospitalisation, the patient fully recovered. Though the stem cell vector can effectively increase the actual dose of virus treatment, the combination strategy of stem cells and oncolytic viruses still needs to be further improved to broaden the delivery of oncolytic DNA viruses.

### Therapeutic evaluation of oncolytic virus for recurrent GBM patients

Since the majority of enrolled patients were recurrent GBM, we extracted the data of rGBM patients and listed the median overall survival as well as the min-OS and max-OS (Table 2). As is illustrated in Table 2, the G47Δ lead in the top-placed rankings with 20.2 months, making it the first and only oncolytic treatment conditional approved for utilization in clinical practice by the local administration. Notably, though the max dose of two trials was the same (3 × 10^9^ PFU), with the increasing repeats of virus injections from 2 to 6, the median overall survival improved significantly from 7.2 months to 20.2 months (Fig. 2d). The mOS rank did not indicated the significant dominance in specific virus type. When it comes to the same oncolytic virus, we supposed that the increasing repeats of administration and the combined treatment might enhance the efficacy of oncolytic virus (Fig. 2e). Currently, the different administrations of viruses did not affect therapeutic benefits significantly based on the limited enrollments.

**Table 2:** The rank of median overall survival of recurrent GBM after oncolytic treatments

Year	Generic name	No.	mKPS	mAge	Administration	Max-dose	mOS	minOS	maxOS
2022	G47Δ	19	80	51	I.T	1 × 10^9^ PFU	20.2	4.2	65.3
2023	DNX-2401	49	90	53	I.T+ pembrolizumab	5 × 10^10^ VP	12.5	2.8	41.6
2018	PVSRIPO	61	90	55	I.T CED	7 × 10^9^ PFU	11.4	3.1	70.4
2017	ParvOryx	18	90	58.5	I.T-excision-I.C/I.V-excision-I.C	5 × 10^9^ PFU	11.2	3.2	40.9
2021	G207	10	–	12.5	I.T + RT	1 × 10^8^ PFU+5 Gy	10.7	3.8	19.4
2018	DNX-2401	33	90	52	I.T/I.T-excision- I.C	3 × 10^10^ VP	9.8	2.3	57.9
2006	NDV-HUJ	11	80	51	I.V.	5.5 × 10^10^ EID50	8	0.75	16.5
2000	HSV1716	8	60	52.5	I.T	1 × 10^5^ PFU	7.5	2	24
2004	HSV1716	4	90	50.5	I.C	1 × 10^5^ PFU	7.5	3	22
2022	G47Δ	13	90	45	I.T	1 × 10^9^ PFU	7.2	3.2	143.9
2009	G207	6	75	54.6	I.T	1.15 × 10^9^ PFU	6.6	2	20.75
2000	G207	16	–	56.5	I.T	3 × 10^9^ PFU	6	2	17
2002	HSV1716	11	80	49	I.T-excision	1 × 10^5^ PFU	6	1	13
2004	ONYX- 015	17	90	55	I.C	1 × 10^10^ PFU	5	1.2	23.4
2014	Reovirus	15	90	51.52	I.T CED	1 × 10^10^ PFU	4.6	3.2	32.9
2022	DNX-2401	19	90	54	I.T CED	3 × 10^10^ PFU	4.3	2.3	93.1
2014	G207	7	90	55	I.T+RT	1.0 × 10^9^ PFU	4.15	1.5	12.8
2008	Reovirus	9	–	–	I.T-excision	7 × 10^8^ PFU	3.25	1.5	15.85

Meanwhile, with the promotion of WHO CNS5, more and more attention has been paid to the molecular typing of glioblastoma patients. Also, it lacks efficient biomarkers for the effectiveness of oncolytic virus therapy. We listed the different molecular type mOS of isocitrate dehydrogenase 1 (IDH1) and O6-methylguanine DNA methyltranferase (MGMT) (Table 3). Todo et al. reported the results that phase II trial of G47Δ indicated there was no differences of oncolytic virus efficacy caused by IDH1 mutation or MGMT expression [10]. It is consistent with the results of Desjardins et al. [30] and Lang et al. [22]. It might show the possibility that the oncolytic virus has consistent efficacy in different IDH or MGMT status.

**Table 3:** The median overall survival of different molecular typing GBM after oncolytic treatments

Generic name	rGBM mOS	No. of patients	mOS		No. of patients	mOS	
		IDH1	wt	mt	MGMT	met	unmet
G47Δ [10]	20.2	wt:13 mt:6	20.9	19.4	met:2 unmet:3 NA:14	16.2	20.2
PVSRIPO [30]	11.4	wt:45 mt:7 NA:9	12.5	13.5	met:14 unmet:36 NA:11	14.3	10.4
ParvOryx [33]	11.2	wt:17 mt:0 NA:1	11.9	–	met:2 unmet:12 NA:4	11	8.9
G207 [20]	10.7	wt:10 mt:0	10.7	–	met:0 unmet:4 NA:6	–	6.9
DNX-2401 [22]	9.8	wt:10 mt:2 NA:21	14.1	11.8	NA:33	–	–
G47Δ [12]	7.2	wt:11 mt:2	11.6	6.2	met:7 unmet:6	11.6	6.9
DNX-2401 [25]	4.3	wt:15 mt:1 NA:3	4.3	93.1	NA:19	–	–

wt represents wild-type; mt represents mutant-type; NA represents none identified; met represents MGMT methylation; unmet represents MGMT unmethylation

## Discussion

Based on the available data, it is evident that oncolytic therapy for malignant gliomas exhibits a favorable safety profile irrespective of the viral type employed. Six virus types have been genetically modified to enhance their oncolytic potential, and they can be broadly categorized into two groups: RNA viruses and DNA viruses. Among these, HSV-1 and adenovirus have predominantly featured in clinical trials of oncolytic virotherapy due to their superior safety profiles and the ease of gene editing technology. In recent years, there has been an increasing focus on developing and utilizing various virus species, particularly RNA viruses such as reovirus, H-1 Parvovirus, PVSRIPO, and NDV. The most notable characteristic of these viruses lies in their ability to traverse the blood–brain barrier, thereby expanding the potential for intravenous administration. However, further evidence is required to substantiate the safety of these novel viral species, address challenges associated with genetic modification, and evaluate their actual efficacy. Moreover, it should be noted that oncolytic viral drugs are typically designed to target a wide range of solid tumors rather than specifically gliomas. Consequently, limited investment has been made in developing oncolytic viruses capable of efficiently crossing the blood–brain barrier.

Based on the analysis of published clinical data, our opinion is that repeated administrations are more effective than single administration in treating malignant glioma with oncolytic virus therapy. The conditionally approved oncolytic product G47Δ has shown significantly increasing efficacy with up to six times of virus injection [10]. However, the mechanism behind it remained breezing, especially the effects of repeated administration on the tumor immune microenvironment. Whether the reinjected virus could arouse the body anti-tumor immune activity was an unsolved question. At the same time, it lacks more rigorous researches to figure out the optimal dose the virus repeated used and the best strategy of delivery approaches. Though the incredible efficacy, six-times surgeries to inject G47Δ is a heavy burden for patients’ both economy and body tolerance. Therefore, other more convenient approaches are on the candidacy such as intraventricular injection, nasal drug delivery, and intravenous injection.

For neurosurgeons, intraventricular administration is a relatively mature method in clinical practice, and there are also widely-used drug delivery products such as Ommaya [35]. However, the viral meningitis seriously hinders the development and application of intraventricular drug delivery. As for intravenous injection, the main obstacle is blood-brain barrier (BBB). Even if the virus species itself has the ability to penetrate the BBB, this will reduce the virus enrichment at the tumor site [29, 31]. Moreover, the current oncolytic viruses developed based on DNA virus, which naturally limit on the capabilities to deliver via nasal or vascular system [36]. Stem cell drug delivery system provides the possible solutions without the completely modified of existing oncolytic viruses [37]. It has been proved the potentials to reduces viral virulence, increases tumor susceptibility and enables intranasal and intravenous administration [38]. Therefore, it might be the trend to solve the repeated administration by the development of stem cell delivery. Moreover, according to the experiences of oncolytic therapies for other tumors, the oncolytic virus combined with other therapies showed synergistic effect and better efficacy [39, 40]. Besides chemotherapy and radiotherapy, increasing research targeted in the combination between oncolytic virus and other immunotherapies including adaptive T-cell transfer and cytokines [41], immune checkpoint inhibitors [26, 42], and antitumor vaccines [40]. Nassiri et al. combined the classical oncolytic adenovirus DNX2401 with Pembrolizumab, and despite considering multivariate factors such as mode of administration and maximum OV dose, rGBM patients experienced a significant increase in median survival, with several achieving long-term survival. This recently published phase II clinical trial further substantiates our perspective on combination strategies, necessitating further exploration of the mechanism underlying pharmacodynamic coordination and investigation into optimal drug combinations and sequencing. The possible mechanism of the synergistic effect is that the other traditional immunotherapies benefit from the shift of tumor immune-microenvironments from ‘cold’ to ‘hot’ caused by the infection and replication of oncolytic viruses [6, 7].

The clinical trials, such as Desjardins et al. and Todo et al., presented some impressive long-term survivors from recurrent GBM. And according to the clinical test results of subjects after treatment, the active viruses do not last more than a few months in the body [10, 12, 24, 43]. Therefore, the lasting anti-tumor immunity might play the critical role in these long-term survivors [44]. Oncolytic virus therapy can sequentially activate innate and adaptive anti-tumor immunity. After viral infection and subsequent oncolysis, a rapid activation of non-specific innate immunity occurs. Infected tumor cells secrete cytokines such as type-I IFN and tumor necrosis factor-α (TNF-α), exposing TAAs and viral PAMPs to the host immune system through oncolysis. This virus-induced cell lysis is recognized as immunogenic cell death (ICD) characterized by the release of danger-associated molecular patterns (DMAPs) including ATP, high-mobility group box 1, and calreticulin. Subsequently, dendritic cells (DCs) are recruited and undergo maturation upon exposure to DAMPs and TAAs. Toll-like receptors on immune cells are activated by DAMPs and PAMPs, leading to the subsequent release of proinflammatory cytokines [e.g., tissue necrosis factor-α (TNF-α), type-I IFN, IL-1β, IL-6, and IL-12] and chemokines. This results in the recruitment and activation of innate immune cells such as neutrophils and NK cells. T cell-mediated immune responses specific to the tumor are crucial for adaptive immunity during OV infection. Depletion of T cells has been shown to result in a lack of anti-tumor efficacy despite persistent OV replication. However, tumor-infiltrating lymphocytes (TILs) are extensively suppressed by components of the tumor microenvironment. Following OV-triggered innate immune responses, antigen-loaded antigen-presenting cells migrate to draining lymph nodes and initiate T cell priming by presenting antigens to naive T cells. Subsequently, production of chemokines that recruit lymphocytes and proinflammatory cytokines during the virus-induced type-I interferon response leads to the recruitment and activation of T cells. Inflammatory factors secreted during OV infection, such as TNF-α, also upregulate selectin expression in endothelial cells, allowing enhanced extravasation of lymphocytes from the vasculature. Then, during T cell infiltration, OV target stromal components and attract neutrophils to alleviate structural barriers in the fibrotic tumor stroma. Both preclinical and clinical studies have shown increased infiltration of cytotoxic T lymphocytes (CTLs) under OV infection, which is associated with a better prognosis [26, 45–47]. Finally, OV reverse the immunosuppressive phenotype of immunoinhibitory cells during conjugation and killing steps by upregulating MHC-I on the tumor cell surface, allowing T cells to escape from immunosuppression and achieve efficient tumor recognition and killing. Therefore, OV participate in all aspects of T cell activation including priming, trafficking, infiltration, activation, and final tumor killing [48].

The idealized tumor immune-microenvironment following oncolytic virus treatment can be divided into two distinct phases. Firstly, it is crucial to suppress the anti-viral immunoreaction in order to facilitate the replication and dissemination of the oncolytic virus. This challenge is particularly evident in viruses such as adenovirus or herpes simplex virus, which may have a high prevalence within the population [49, 50]. Furthermore, repeated administration of the virus might exacerbate this phenomenon. Secondly, after tumor cell lysis and exposure to tumor antigens, a robust and enduring anti-tumor immunity should be induced [51, 52]. Therefore, local immunity can act as a double-edged sword. Further investigations into the underlying mechanisms and genetic modifications of viruses are necessary for effective control over the immune-microenvironment.

As oncolytic virotherapy is still in its early stages, two practical issues for future clinical use are biomarkers and timing of administration. Unlike other chemotherapy drugs, there is a lack of suitable markers to evaluate the efficacy potential of oncolytic therapy [53]. As we primarily demonstrated from the mOS of rGBM patients with different molecular subtype (IDH1 and MGMT), the common genotyping is not a good predictor of oncolytic virus efficacy. However, some biomarkers are depicting great potentials including IFN signaling elements, cGAS–STING, retinoic acid-inducible gene I (RIG-I), and various Toll-like receptors (TLRs) [54]. In future clinical trials, the detection and analysis of these possible markers deserve more attentions. Also, the majority of studies have focused on recurrent GBM patients, while few data and comparisons applied to initial glioma patients.

## Conclusion

The past 20 years witness the rapid development of OV for malignant glioma, and the first oncolytic product conditional approved in Japan brightens the future of OV developments. Besides direct tumor lysis, oncolytic therapy arouses the anti-tumor immunity and shows the potential of long-term treatment for tumor. According to the systematic review of clinical trials for malignant glioma treatments, we glanced the safety and developments of OV. In the future, the repeated administrations and the combination with other immunotherapies are required more explorations. The next generation of the existing virus by gene editing or the stem cell delivery system were urged to be developed. Meanwhile, we advocate for the attention and disclosure of tumor molecular or genotyping information in subsequent clinical trials on ovarian cancer (OV) to facilitate the analysis of biomarkers that can predict treatment response, a critical factor for the successful implementation of OV therapy.

In a nutshell, OV for malignant glioma shows great prospects in the future and promotes significantly development of cancer therapy.

## References

[1] Tan AC, Ashley DM, Lopez GY, Malinzak M, Friedman HS, Khasraw M (2020). Management of glioblastoma: state of the art and future directions. CA Cancer J Clin.

[2] Sanmamed MF, Chen L (2019). A paradigm shift in cancer Immunotherapy: from enhancement to normalization. Cell.

[3] Stojdl DF, Lichty B, Knowles S, Marius R, Atkins H, Sonenberg N, Bell JC (2000). Exploiting tumor-specific defects in the interferon pathway with a previously unknown oncolytic virus. Nat Med.

[4] Adair RA, Roulstone V, Scott KJ, Morgan R, Nuovo GJ, Fuller M, Beirne D, West EJ, Jennings VA, Rose A, Kyula J, Fraser S, Dave R, Anthoney DA, Merrick A, Prestwich R, Aldouri A, Donnelly O, Pandha H, Coffey M, Selby P, Vile R, Toogood G, Harrington K, Melcher AA (2012). Cell carriage, delivery, and selective replication of an oncolytic virus in tumor in patients. Sci Transl Med.

[5] Shi T, Song X, Wang Y, Liu F, Wei J (2020). Combining oncolytic viruses with cancer immunotherapy: establishing a new generation of cancer treatment. Front Immunol.

[6] Duan Q, Zhang H, Zheng J, Zhang L (2020). Turning cold into hot: firing up the tumor microenvironment. Trends in Cancer.

[7] Gujar S, Pol JG, Kroemer G (2018). Heating it up: oncolytic viruses make tumors ‘hot’ and suitable for checkpoint blockade immunotherapies. Oncoimmunology.

[8] Mantwill K, Naumann U, Seznec J, Girbinger V, Lage H, Surowiak P, Beier D, Mittelbronn M, Schlegel J, Holm PS (2013). YB-1 dependent oncolytic adenovirus efficiently inhibits tumor growth of glioma cancer stem like cells. J Transl Med.

[9] Jiang H, White EJ, Gomez-Manzano C, Fueyo J (2008). Adenovirus’s last trick: you say lysis, we say autophagy. Autophagy.

[10] Todo T, Ito H, Ino Y, Ohtsu H, Ota Y, Shibahara J, Tanaka M (2022). Intratumoral oncolytic herpes virus G47∆ for residual or recurrent glioblastoma: a phase 2 trial. Nat Med.

[11] Page MJ, McKenzie JE, Bossuyt PM, Boutron I, Hoffmann TC, Mulrow CD, Shamseer L, Tetzlaff JM, Akl EA, Brennan SE, Chou R, Glanville J, Grimshaw JM, Hrobjartsson A, Lalu MM, Li T, Loder EW, Mayo-Wilson E, McDonald S, McGuinness LA, Stewart LA, Thomas J, Tricco AC, Welch VA, Whiting P, Moher D (2021). The PRISMA 2020 statement: an updated guideline for reporting systematic reviews. Rev Esp Cardiol (Engl Ed).

[12] Todo T, Ino Y, Ohtsu H, Shibahara J, Tanaka M (2022). A phase I/II study of triple-mutated oncolytic herpes virus G47 in patients with progressive glioblastoma. Nat Commun.

[13] Li J, Meng Q, Zhou X, Zhao H, Wang K, Niu H, Wang Y (2022). Gospel of malignant glioma: oncolytic virus therapy. Gene.

[14] Markert JM, Medlock MD, Rabkin SD, Gillespie GY, Todo T, Hunter WD, Palmer CA, Feigenbaum F, Tornatore C, Tufaro F, Martuza RL (2000). Conditionally replicating herpes simplex virus mutant, G207 for the treatment of malignant glioma: results of a phase I trial. Gene Ther.

[15] Rampling R, Cruickshank G, Papanastassiou V, Nicoll J, Hadley D, Brennan D, Petty R, MacLean A, Harland J, McKie E, Mabbs R, Brown M (2000). Toxicity evaluation of replication-competent herpes simplex virus (ICP 34.5 null mutant 1716) in patients with recurrent malignant glioma. Gene Ther.

[16] Papanastassiou V, Rampling R, Fraser M, Petty R, Hadley D, Nicoll J, Harland J, Mabbs R, Brown M (2002). The potential for efficacy of the modified (ICP 34.5(-)) herpes simplex virus HSV1716 following intratumoural injection into human malignant glioma: a proof of principle study. Gene Ther.

[17] Harrow S, Papanastassiou V, Harland J, Mabbs R, Petty R, Fraser M, Hadley D, Patterson J, Brown SM, Rampling R (2004). HSV1716 injection into the brain adjacent to tumour following surgical resection of high-grade glioma: safety data and long-term survival. Gene Ther.

[18] Markert JM, Liechty PG, Wang W, Gaston S, Braz E, Karrasch M, Nabors LB, Markiewicz M, Lakeman AD, Palmer CA, Parker JN, Whitley RJ, Gillespie GY (2009). Phase ib trial of mutant herpes simplex virus G207 inoculated pre-and post-tumor resection for recurrent GBM. Mol Therapy J Am Soc Gene Therapy.

[19] Markert JM, Razdan SN, Kuo H-C, Cantor A, Knoll A, Karrasch M, Nabors LB, Markiewicz M, Agee BS, Coleman JM, Lakeman AD, Palmer CA, Parker JN, Whitley RJ, Weichselbaum RR, Fiveash JB, Gillespie GY (2014). A phase 1 trial of oncolytic HSV-1, G207, given in combination with radiation for recurrent GBM demonstrates safety and radiographic responses. Mol Therapy J Am Soc Gene Therapy.

[20] Friedman GK, Johnston JM, Bag AK, Bernstock JD, Li R, Aban I, Kachurak K, Nan L, Kang K-D, Totsch S, Schlappi C, Martin AM, Pastakia D, McNall-Knapp R, Farouk Sait S, Khakoo Y, Karajannis MA, Woodling K, Palmer JD, Osorio DS, Leonard J, Abdelbaki MS, Madan-Swain A, Atkinson TP, Whitley RJ, Fiveash JB, Markert JM, Gillespie GY (2021). Oncolytic HSV-1 G207 immunovirotherapy for pediatric high-grade gliomas. N Engl J Med.

[21] Chiocca EA, Abbed KM, Tatter S, Louis DN, Hochberg FH, Barker F, Kracher J, Grossman SA, Fisher JD, Carson K, Rosenblum M, Mikkelsen T, Olson J, Markert J, Rosenfeld S, Nabors LB, Brem S, Phuphanich S, Freeman S, Kaplan R, Zwiebel J (2004). A phase I open-label, dose-escalation, multi-institutional trial of injection with an E1B-attenuated adenovirus, ONYX-015, into the peritumoral region of recurrent malignant gliomas, in the adjuvant setting. Mol Ther.

[22] Lang FF, Conrad C, Gomez-Manzano C, Yung WKA, Sawaya R, Weinberg JS, Prabhu SS, Rao G, Fuller GN, Aldape KD, Gumin J, Vence LM, Wistuba I, Rodriguez-Canales J, Villalobos PA, Dirven CMF, Tejada S, Valle RD, Alonso MM, Ewald B, Peterkin JJ, Tufaro F, Fueyo J (2018). Phase I study of DNX-2401 (Delta-24-RGD) oncolytic adenovirus: replication and immunotherapeutic effects in recurrent malignant glioma. J Clin Oncol.

[23] Fares J, Ahmed AU, Ulasov IV, Sonabend AM, Miska J, Lee-Chang C, Balyasnikova IV, Chandler JP, Portnow J, Tate MC, Kumthekar P, Lukas RV, Grimm SA, Adams AK, Hébert CD, Strong TV, Amidei C, Arrieta VA, Zannikou M, Horbinski C, Zhang H, Burdett KB, Curiel DT, Sachdev S, Aboody KS, Stupp R, Lesniak MS (2021). Neural stem cell delivery of an oncolytic adenovirus in newly diagnosed malignant glioma: a first-in-human, phase 1, dose-escalation trial. Lancet Oncol.

[24] Gállego Pérez-Larraya J, Garcia-Moure M, Labiano S, Patiño-García A, Dobbs J, Gonzalez-Huarriz M, Zalacain M, Marrodan L, Martinez-Velez N, Puigdelloses M, Laspidea V, Astigarraga I, Lopez-Ibor B, Cruz O, Oscoz Lizarbe M, Hervas-Stubbs S, Alkorta-Aranburu G, Tamayo I, Tavira B, Hernandez-Alcoceba R, Jones C, Dharmadhikari G, Ruiz-Moreno C, Stunnenberg H, Hulleman E, van der Lugt J, Idoate M, Diez-Valle R, Esparragosa Vázquez I, Villalba M, de Andrea C, Núñez-Córdoba JM, Ewald B, Robbins J, Fueyo J, Gomez-Manzano C, Lang FF, Tejada S, Alonso MM (2022). Oncolytic DNX-2401 virus for pediatric diffuse intrinsic pontine glioma. N Engl J Med.

[25] van Putten EHP, Kleijn A, van Beusechem VW, Noske D, Lamers CHJ, de Goede AL, Idema S, Hoefnagel D, Kloezeman JJ, Fueyo J, Lang FF, Teunissen CE, Vernhout RM, Bakker C, Gerritsen W, Curiel DT, Vulto A, Lamfers MLM, Dirven CMF (2022). Convection enhanced delivery of the oncolytic adenovirus delta24-RGD in patients with recurrent GBM: a phase I clinical trial including correlative studies. Clin Cancer Res.

[26] Nassiri F, Patil V, Yefet LS, Singh O, Liu J, Dang RMA, Yamaguchi TN, Daras M, Cloughesy TF, Colman H, Kumthekar PU, Chen CC, Aiken R, Groves MD, Ong SS, Ramakrishna R, Vogelbaum MA, Khagi S, Kaley T, Melear JM, Peereboom DM, Rodriguez A, Yankelevich M, Nair SG, Puduvalli VK, Aldape K, Gao A, Lopez-Janeiro A, de Andrea CE, Alonso MM, Boutros P, Robbins J, Mason WP, Sonabend AM, Stupp R, Fueyo J, Gomez-Manzano C, Lang FF, Zadeh G (2023). Oncolytic DNX-2401 virotherapy plus pembrolizumab in recurrent glioblastoma: a phase 1/2 trial. Nat Med.

[27] Forsyth P, Roldan G, George D, Wallace C, Palmer CA, Morris D, Cairncross G, Matthews MV, Markert J, Gillespie Y, Coffey M, Thompson B, Hamilton M (2008). A phase I trial of intratumoral administration of reovirus in patients with histologically confirmed recurrent malignant gliomas. Mol Ther.

[28] Kicielinski KP, Chiocca EA, Yu JS, Gill GM, Coffey M, Markert JM (2014). Phase 1 clinical trial of intratumoral reovirus infusion for the treatment of recurrent malignant gliomas in adults. Mol Therapy J Am Soc Gene Therapy.

[29] Geletneky K, Hajda J, Angelova AL, Leuchs B, Capper D, Bartsch AJ, Neumann JO, Schöning T, Hüsing J, Beelte B, Kiprianova I, Roscher M, Bhat R, von Deimling A, Brück W, Just A, Frehtman V, Löbhard S, Terletskaia-Ladwig E, Fry J, Jochims K, Daniel V, Krebs O, Dahm M, Huber B, Unterberg A, Rommelaere J (2017). Oncolytic H-1 parvovirus shows safety and signs of immunogenic activity in a first phase I/IIa glioblastoma trial. Mol Ther.

[30] Desjardins A, Gromeier M, Herndon JE, Beaubier N, Bolognesi DP, Friedman AH, Friedman HS, McSherry F, Muscat AM, Nair S, Peters KB, Randazzo D, Sampson JH, Vlahovic G, Harrison WT, McLendon RE, Ashley D, Bigner DD (2018). Recurrent glioblastoma treated with recombinant poliovirus. N Engl J Med.

[31] Freeman AI, Zakay-Rones Z, Gomori JM, Linetsky E, Rasooly L, Greenbaum E, Rozenman-Yair S, Panet A, Libson E, Irving CS, Galun E, Siegal T (2006). Phase I/II trial of intravenous NDV-HUJ oncolytic virus in recurrent glioblastoma multiforme. Mol Ther.

[32] Pérez-Larraya JG, Garcia-Moure M, Labiano S, Patiño-García A, Dobbs J, Gonzalez-Huarriz M, Zalacain M, Marrodan L, Martinez-Velez N, Puigdelloses M, Laspidea V, Astigarraga I, Lopez-Ibor B, Cruz O, Lizarbe MO, Hervas-Stubbs S, Alkorta-Aranburu G, Tamayo I, Tavira B, Hernandez-Alcoceba R, Jones C, Dharmadhikari G, Ruiz-Moreno C, Stunnenberg H, Hulleman E, van der Lugt J, Idoate M, Diez-Valle R, Vázquez IE, Villalba M, de Andrea C, Núñez-Córdoba JM, Ewald B, Robbins J, Fueyo J, Gomez-Manzano C, Lang FF, Tejada S, Alonso MM (2022). Oncolytic DNX-2401 virus for pediatric diffuse intrinsic pontine glioma. N Engl J Med.

[33] Geletneky K, Hajda J, Angelova AL, Leuchs B, Capper D, Bartsch AJ, Neumann J-O, Schöning T, Hüsing J, Beelte B, Kiprianova I, Roscher M, Bhat R, von Deimling A, Brück W, Just A, Frehtman V, Löbhard S, Terletskaia-Ladwig E, Fry J, Jochims K, Daniel V, Krebs O, Dahm M, Huber B, Unterberg A, Rommelaere J (2017). Oncolytic H-1 parvovirus shows safety and signs of immunogenic activity in a first phase I/IIa glioblastoma trial. Mol Therapy J Am Soc Gene Therapy.

[34] Desjardins A, Gromeier M, Herndon JE, Beaubier II, Bolognesi N, Friedman DP, Friedman AH, McSherry HS, Muscat F, Nair AM, Peters S, Randazzo KB, Sampson D, Vlahovic JH, Harrison G, McLendon WT, Ashley RE, Bigner D (2018). Recurrent glioblastoma treated with recombinant poliovirus. N Engl J Med.

[35] Wilson R, Osborne C, Halsey C (2018). The use of ommaya reservoirs to deliver central nervous system-directed chemotherapy in childhood acute lymphoblastic leukaemia. Paediatr Drugs.

[36] Chen W, Yao S, Wan J, Tian Y, Huang L, Wang S, Akter F, Wu Y, Yao Y, Zhang X (2021). BBB-crossing adeno-associated virus vector: an excellent gene delivery tool for CNS disease treatment. J Control Release.

[37] Bahreyni A, Ghorbani E, Fuji H, Ryzhikov M, Khazaei M, Erfani M, Avan A, Hassanian SM, Azadmanesh K (2019). Therapeutic potency of oncolytic virotherapy-induced cancer stem cells targeting in brain tumors, current status, and perspectives. J Cell Biochem.

[38] Batalla-Covello J, Ngai HW, Flores L, McDonald M, Hyde C, Gonzaga J, Hammad M, Gutova M, Portnow J, Synold T, Curiel DT, Lesniak MS, Aboody KS, Mooney R (2021). Multiple treatment cycles of neural stem cell delivered oncolytic adenovirus for the treatment of glioblastoma. Cancers.

[39] Zhou Z, Tian J, Zhang W, Xiang W, Ming Y, Chen L, Zhou J (2021). Multiple strategies to improve the therapeutic efficacy of oncolytic herpes simplex virus in the treatment of glioblastoma (review). Oncol Lett.

[40] Tang B, Guo ZS, Bartlett DL, Yan DZ, Schane CP, Thomas DL, Liu J, McFadden G, Shisler JL, Roy EJ (2020). Synergistic combination of oncolytic virotherapy and immunotherapy for glioma. Clin Cancer Res.

[41] Ajina A, Maher J (2019). Synergistic combination of oncolytic virotherapy with CAR T-cell therapy. Prog Mol Biol Transl Sci.

[42] Samson A, Scott KJ, Taggart D, West EJ, Wilson E, Nuovo GJ, Thomson S, Corns R, Mathew RK, Fuller MJ, Kottke TJ, Thompson JM, Ilett EJ, Cockle JV, van Hille P, Sivakumar G, Polson ES, Turnbull SJ, Appleton ES, Migneco G, Rose AS, Coffey MC, Beirne DA, Collinson FJ, Ralph C, Alan Anthoney D, Twelves CJ, Furness AJ, Quezada SA, Wurdak H, Errington-Mais F, Pandha H, Harrington KJ, Selby PJ, Vile RG, Griffin SD, Stead LF, Short SC, Melcher AA (2018). Intravenous delivery of oncolytic reovirus to brain tumor patients immunologically primes for subsequent checkpoint blockade. Sci Transl Med.

[43] Geletneky K, Hajda J, Angelova AL, Leuchs B, Capper D, Bartsch AJ, Neumann J-O, Schoening T, Huesing J, Beelte B, Kiprianova I, Roscher M, Bhat R, von Deimling A, Brueck W, Just A, Frehtman V, Loebhard S, Terletskaia-Ladwig E, Fry J, Jochims K, Daniel V, Krebs O, Dahm M, Huber B, Unterberg A, Rommelaere J (2017). Oncolytic H-1 parvovirus shows safety and signs of immunogenic activity in a first phase I/IIa glioblastoma trial. Mol Ther.

[44] Ene CI, Fueyo J, Lang FF (2021). Delta-24 adenoviral therapy for glioblastoma: evolution from the bench to bedside and future considerations. Neurosurg Focus.

[45] Chon HJ, Lee WS, Yang H, Kong SJ, Lee NK, Moon ES, Choi J, Han EC, Kim JH, Ahn JB, Kim JH, Kim C (2019). Tumor microenvironment remodeling by intratumoral oncolytic vaccinia virus enhances the efficacy of immune-checkpoint blockade. Clin Cancer Res.

[46] Ribas A, Dummer R, Puzanov I, VanderWalde A, Andtbacka RHI, Michielin O, Olszanski AJ, Malvehy J, Cebon J, Fernandez E, Kirkwood JM, Gajewski TF, Chen L, Gorski KS, Anderson AA, Diede SJ, Lassman ME, Gansert J, Hodi FS, Long GV (2017). Oncolytic virotherapy promotes intratumoral T cell infiltration and improves anti-PD-1 immunotherapy. Cell.

[47] Garcia-Carbonero R, Salazar R, Duran I, Osman-Garcia I, Paz-Ares L, Bozada JM, Boni V, Blanc C, Seymour L, Beadle J, Alvis S, Champion B, Calvo E, Fisher K (2017). Phase 1 study of intravenous administration of the chimeric adenovirus enadenotucirev in patients undergoing primary tumor resection. J Immunother Cancer.

[48] Twumasi-Boateng K, Pettigrew JL, Kwok YYE, Bell JC, Nelson BH (2018). Oncolytic viruses as engineering platforms for combination immunotherapy. Nat Rev Cancer.

[49] Meisen WH, Wohleb ES, Jaime-Ramirez AC, Bolyard C, Yoo JY, Russell L, Hardcastle J, Dubin S, Muili K, Yu J, Caligiuri M, Godbout J, Kaur B (2015). The impact of macrophage- and microglia-secreted TNFalpha on oncolytic HSV-1 therapy in the glioblastoma tumor microenvironment. Clin Cancer Res.

[50] Saha D, Wakimoto H, Rabkin SD (2016). Oncolytic herpes simplex virus interactions with the host immune system. Curr Opin Virol.

[51] Chiocca EA, Rabkin SD (2014). Oncolytic viruses and their application to cancer immunotherapy. Cancer Immunol Res.

[52] Saha D, Martuza RL, Rabkin SD (2017). Macrophage polarization contributes to glioblastoma eradication by combination immunovirotherapy and immune checkpoint blockade. Cancer Cell.

[53] Zeng J, Li X, Sander M, Zhang H, Yan G, Lin Y (2021). Oncolytic viro-immunotherapy: an emerging option in the treatment of gliomas. Front Immunol.

[54] Matveeva OV, Chumakov PM (2018). Defects in interferon pathways as potential biomarkers of sensitivity to oncolytic viruses. Rev Med Virol.

